# Association between lean body mass to visceral fat mass ratio and bone mineral density in United States population: a cross-sectional study

**DOI:** 10.1186/s13690-023-01190-4

**Published:** 2023-10-06

**Authors:** Longti Li, Huiqin Zhong, Ya Shao, Xu Zhou, Yu Hua, Maoqian Chen

**Affiliations:** 1 Department of Nursing, TaiHe Hospital, Hubei University of Medicine, Shiyan, China; 2 Department of Gastroenterology, TaiHe Hospital, Hubei University of Medicine, Shiyan, China; 3 Health Management Center, TaiHe Hospital, Hubei University of Medicine, Shiyan, China

**Keywords:** Bone mineral density, Lean body mass, NHANES, Visceral fat mass

## Abstract

**Background:**

Studies have explored the correlation between body composition and bone mineral density (BMD), but there has yet to be a consensus. Thus, the present study aims to comprehensively investigate the association between lean body mass, adipose tissue, and BMD.

**Methods:**

We conducted a cross-sectional study using data from the National Health and Nutrition Examination Survey (NHANES) (2011–2018) with 11,227 subjects. Multiple linear regression, smoothed curve fitting, threshold, and saturation effect analysis were used to explore the association between lean body mass, visceral fat mass, and BMD. Also, we used the lean body mass to visceral fat mass ratio (Log LM/VFM) as a proxy variable to analyze its association with BMD alone.

**Results:**

After adjusting for potential confounding factors, the results showed a positive correlation between lean mass and total BMD (for continuous: β = 0.078, P < 0.001; for quartile: β = 0.138, P < 0.001), while visceral fat mass was negatively correlated (for continuous: β = -0.027, P < 0.001; for quartile: β = -0.065, P < 0.001). A positive correlation was observed when the alternative variable Log LM/VFM was analyzed separately for its association with BMD (for continuous: β = 0.034, P < 0.001; for quartile: β = 0.084, P < 0.001). In addition, subgroup analyses for gender, age, body mass index, hypertension, and diabetes showed that all subgroups except the diabetes subgroup showed a substantial degree of robustness (P < 0.05). The smoothed curve fitting showed a nonlinear relationship between Log LM/VFM and BMD, and there was a threshold effect with a critical value of 2.60.

**Conclusion:**

Maintaining a proper ratio of lean body mass and visceral fat mass is beneficial for increasing BMD.

**Table:** 

**Text box 1. Contributions to the literature**
Osteoporosis dramatically threatens the health status of the middle-aged and elderly population, with more than 9 million worldwide suffering from osteoporotic fractures yearly.
Bone mineral density (BMD) is an essential indicator for evaluating osteoporosis. Studies have shown that obesity is strongly associated with BMD, but conflicting results have emerged among existing studies.
Lean body mass to visceral fat mass ratio was positively and nonlinearly correlated with BMD, and there was a threshold effect.
There is an urgent need for appropriate public health policies to guide people to maintain an appropriate muscle-fat ratio to improve BMD.

## Introduction

Osteoporosis poses a significant threat to population health and results in a considerable socioeconomic burden. In the United States, the prevalence of osteoporosis is high, with 12.6% of the population over 50 years of age affected [1]. Furthermore, fractures afflict 1.5 million people annually [2]. To assess bone health status, a crucial parameter for osteoporosis diagnosis, fracture risk prediction, and treatment efficacy evaluation is bone mineral density (BMD), which measures bone mass content and density [3]. Lately, there has been a growing research interest in bone metabolism-associated risk and protective factors, particularly obesity.

Several studies have concluded that obesity has a protective effect on BMD. A study of 2903 older adults by Zhang et al. [4] showed a significant positive correlation between body mass index (BMI) and BMD, and a study of 4056 adolescents by Wang et al. [5] showed similar findings. Two systematic reviews support this finding demonstrating that obese individuals have heightened BMD and improved bone microarchitecture [6, 7]. However, recent discoveries have challenged this view revealing that obesity might increase the incidence of fractures [8, 9]. Consequently, the “obesity paradox” has been coined, which proposes that adipose tissue and bone cells mutually interact and that obesity has both protective and detrimental effects on bone metabolism [10]. A Mendelian randomization analysis conducted by Du et al. [11] using data from the UK Biobank database, which included 265,627 individuals, revealed a negative association between hip circumference adjusted for BMI and BMD. Conversely, the waist-to-hip ratio was found to be positively associated with BMD. These conflicting results suggest that the relationship between obesity and BMD needs further clarification.

Previous studies on obesity and BMD have mainly assessed the degree of obesity through simple measures such as BMI, waist circumference, and waist-to-hip ratio, especially the former. However, these methods do not provide a precise enough evaluation of the degree of obesity or a comprehensive reflection of body fat distribution [12]. Therefore, researchers have tried to evaluate its association with BMD by more accurately evaluating the distribution of human adipose tissue, such as body fat percentage, total fat mass, and abdominal or visceral fat mass. Nevertheless, the conclusions of these studies were conflicting. A survey by Zhu et al. [13] of 4865 Australian Caucasians aged 45–70 showed that visceral adipose tissue was inversely associated with BMD. Fan et al.’s [14] survey of 357 non-obese postmenopausal women over 60 years old in China showed that total fat mass was positively correlated with BMD, and the android-to-gynoid fat ratio was negatively correlated with BMD. The heterogeneity among these study populations and indicators used to evaluate obesity were not identical in these studies, limiting the results’ consistency.

Not only that, but researchers have explored the combined effect of the adipose tissue and muscle with BMD. A meta-analysis of middle-aged and older adults over 50 showed that the mean BMD of the femoral neck was lower in people with sarcopenic obesity compared to those with simple obesity but was higher than the population of sarcopenia alone [15]. Limited evidence suggests differences in the health effects of adipose tissue in different parts of the body. Subcutaneous and lower body adipose tissue may have a protective effect on bone. A case-control study of 169 hip fractures showed that reduced subcutaneous adipose tissue was associated with an increased risk of fracture [16]. In contrast, two other studies have shown that visceral fat or abdominal adipose tissue accumulation may adversely affect BMD [17, 18].

Current studies have separately investigated the relationship between lean body mass and adipose tissue with BMD. However, it is crucial to acknowledge that these two components are not only closely interconnected in the human body but may also have distinct impacts on human metabolic health. Exploring the association of both with BMD in isolation provides only a partial picture of the relationship between the variables. Given conflicting reports of the existing studies, we will examine the associations between lean body mass, visceral fat mass, and BMD in isolation. At the same time, we used a novel marker, lean body mass to visceral fat mass ratio, to reflect more comprehensively the combined effect of muscle and adipose tissue distribution on BMD.

## Materials and methods

### Study design and participants

**Fig. 1:**
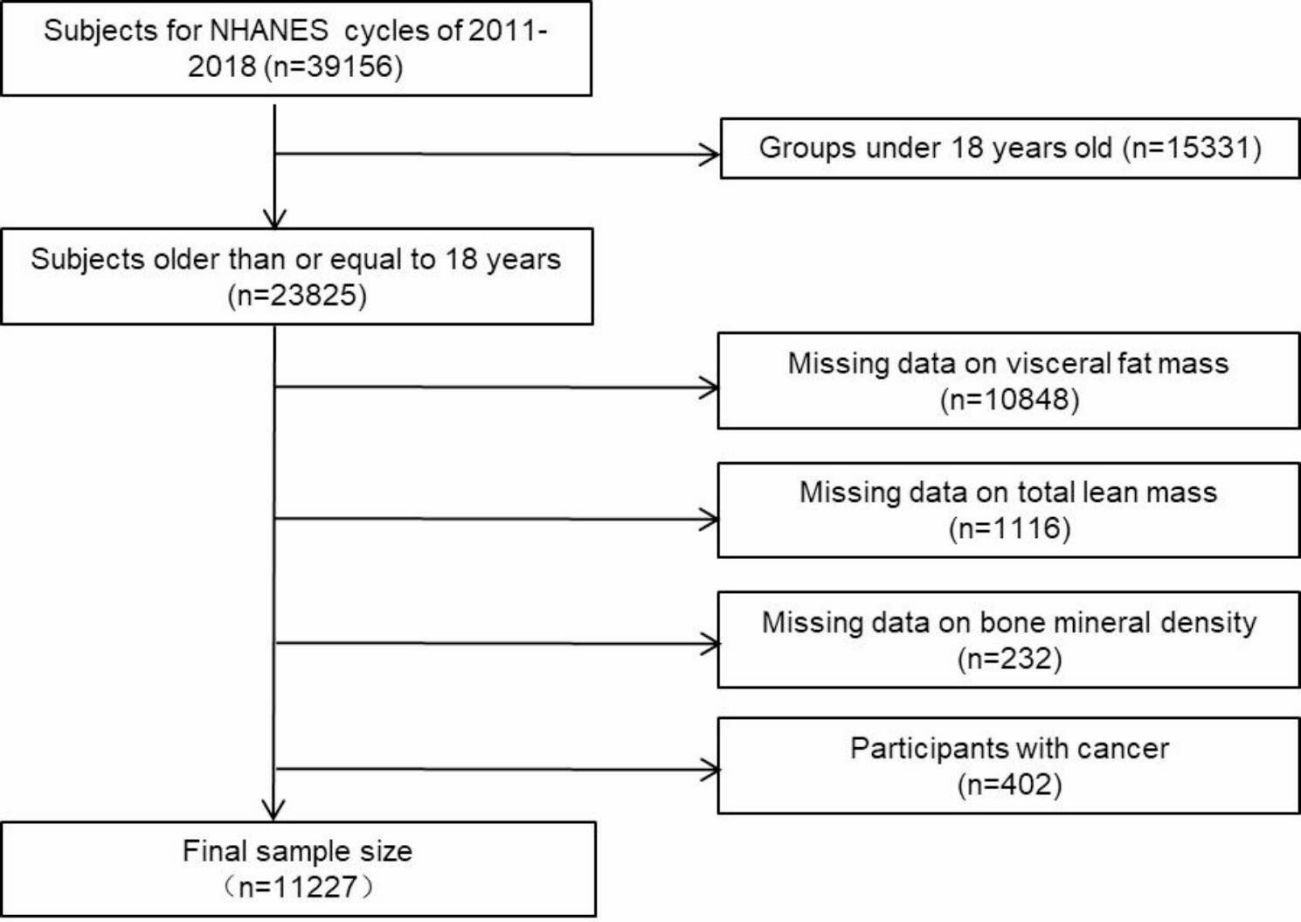
The flowchart describes the process of enrolling study subjects in the National Health and Nutrition Examination Survey 2011–2018 to explore associations between lean body mass, visceral fat mass, and lean body mass to visceral fat ratio with bone mineral density

This study utilized a cross-sectional design and obtained all data from the National Health and Nutrition Examination Survey (NHANES), a comprehensive assessment survey conducted biennially to evaluate the health and nutritional status of the general U.S. population through a multi-stage probability sample. Data were collected through interviews, physical examinations, and laboratory tests [19]. Four cycles of data from NHANES 2011–2018 were comprised in this study, with 39,156 participants in these cycles. We excluded those younger than 18 years of age, those with tumors, and those missing information on lean body mass, visceral fat mass, and BMD, resulting in a total of 11,227 individuals being included. The National Center for Health Statistics Institutional Review Board of the United States approved the survey, and informed consent was obtained from all participants before implementation. The screening process used to select the study population is shown in Fig. 1.

### Exposure and outcome variables

The exposure variables in this study were total lean body mass (excluding bone mineral content), visceral fat mass, and the ratio of lean body mass to visceral fat mass, while the outcome variable was BMD. All these variables were measured via Dual-energy X-ray absorptiometry, a standard method for gauging body composition and BMD status that provides accurate measures and low radiation exposure. However, there were some exclusions, i.e., pregnancy, past X-ray contrast (barium) exposure within the previous week, and weighing over 450 lbs. Certified and skilled radiographers conducted all the scans. An anthropomorphic spine model by Hologic underwent a daily scan to ensure the densitometer was precisely calibrated. To ensure the measurements’ dependability, quality control personnel reviewed every participant scan examined, which amounted to 100%. The procedures manual describes specific equipment, supplies, materials, protocols, and quality control measures [20].

### Covariates

To minimize potential confounders, we analyzed various general demographic characteristics of the study population, such as gender, age, race, marital status, education, poverty ratio of family income (PIR), BMI, physical activity, smoking and drinking habits. According to the current guideline [21], physical activity levels were categorized as active, inactive, and completely inactive. “Active” was defined as physical activity that involved high-intensity exercise for more than 75 min or a moderate-intensity routine for more than 150 min per week. “Inactive” included physical activity that did not meet the abovementioned criteria. “Completely inactive” were those who did not participate in physical activity. At the same time, we also collected metabolic equivalent (MET) scores from the participants, which were calculated based on the intensity of each type of activity and the corresponding activity scores [22].

Smoking status was categorized as never, former, and current. Non-smoking was defined as someone who never smoked more than 100 cigarettes in their lifetime. A former smoker was defined as someone who had smoked more than 100 cigarettes in their lifetime but had currently quit, and a current smoker was defined as someone who smoked more than 100 cigarettes and was presently smoking [23]. Alcohol consumption was determined by a cut-off of > 12 drinks per year, and no alcohol consumption was defined as consuming less than 12 drinks per year [24].

In addition, we assessed the impact of underlying chronic diseases such as hypertension, diabetes, and thyroid problem. Hypertension was described as a prior diagnosis of high blood pressure or antihypertensive medication use or as having blood pressure levels of systolic blood pressure ≥ 140 mmHg or diastolic blood pressure ≥ 90 mmHg measured on three different days [25]. Diabetes was defined as the previous diagnosis, use of glucose-lowering medication or insulin, fasting blood glucose levels greater than or equal to 126 mg/dL, 2-hour OGTT ≥ 200 mg/dL, or glycosylated hemoglobin ≥ 6.5% [26]. Thyroid disease was evaluated using the following question: “Has a doctor or other health professional ever told you had a thyroid problem.“

We also assessed participants’ Vitamin D and Calcium intake and dietary supplements. Such data were estimated from two day 24 h dietary interviews, and we used the mean of the data when available. Furthermore, we evaluated the effects of relevant blood markers such as total cholesterol, alanine aminotransferase, and serum uric acid.

### Statistical analysis

Continuous variables are expressed as mean ± standard deviation or median and interquartile range, and categorical variables are depicted as percentages. Mobile Examination Centre (MEC) test weights were employed in the analysis to improve data representativeness. In cases where there is missing data for covariates, our approach differs depending on the variable type. The missing data is replaced with an ‘unclear’ group for categorical variables. We use dummy variables to indicate missingness and include them in the regression model along with the original variables for continuous variables. Multiple linear regression analysis investigated the relationship between exposure and outcome variables. To assess the association between lean body mass and visceral fat mass in combination with BMD, the lean body mass to visceral fat mass ratio was used as a separate exposure variable and was log10 transformed to create normally distributed estimates (Log LM/VFM). The median of each quartile was used to test the linear trend across quartiles.

The research findings include both crude regression estimates and estimates that have been adjusted for covariates. We selected adjusted confounders based on clinical importance for the outcome variable. In the Crude Model, we did not adjust for any variables; in Model 1, we adjusted for sex, age, and race; in Model 2, we further adjusted for the variables in Model 1 as well as marital status, education, PIR, BMI, activity, MET scores, smoking, alcohol, hypertension, diabetes, thyroid disease, serum vitamin D, total cholesterol, alanine aminotransferase, total calcium, serum uric acid, vitamin D intake, vitamin D supplements, calcium intake, and calcium supplements.

We used generalized additive models to assess the dose-response relationship between exposure variables and BMD. In contrast, we used a two-stage linear regression model to analyze the threshold effect of LogLM/VFM on BMD. In addition, separate stratified analyses by sex, age, BMI, hypertension, and diabetes were performed to verify the robustness of the results. The data were analyzed using the R (version 3.4.3, http://www.R-project.org) and Empower software (version 2.0, http://www.empowerstats.com).

## Results

### Characteristics of participants

Table 1 displays the fundamental characteristics of the studied population. Of the 11,227 participants, 5,722 were men, and 5,505 were women, with an average age of 37.23 ± 12.34 years. The participants were segregated into four groups according to their BMD quartiles. The comparison between groups revealed substantial differences concerning sex, age, marital status, ethnic background, educational attainment, PIR, physical activity level, smoking status, alcohol consumption, hypertension, and thyroid disease. Those within the highest BMD quartile presented comparatively higher BMI, MET scores, vitamin D and calcium intake, total calcium, alanine transaminase, total lean mass, serum uric acid, and LogLM/VFM, with lower visceral fat mass, serum vitamin D, blood cholesterol levels and calcium supplements.

**Table 1:** Characteristics of the study population by bone mineral density quartiles in the National Health and Nutrition Examination Survey 2011–2018

Characteristic	Total bone mineral density				
	Q1 ≤ 1.03	Q2 1.04–1.10	Q3 1.11–1.17	Q4 ≥ 1.18	P-value
**No**	2806	2805	2798	2818	
**Gender, n (%)**					< 0.001
Male	920 (32.79)	1253 (44.67)	1551 (55.43)	1998 (70.90)	
Female	1886 (67.21)	1552 (55.33)	1247 (44.57)	820 (29.10)	
**Age (mean ± SD, year)**	38.93 ± 13.45	36.66 ± 12.24	36.62 ± 11.70	36.72 ± 11.76	< 0.001
**Race, n (%)**					< 0.001
Mexican American	538 (19.17)	437 (15.58)	463 (16.55)	301 (10.68)	
Non-Hispanic White	888 (31.65)	1012 (36.08)	972 (34.74)	842 (29.88)	
Non-Hispanic Black	278 (9.91)	427 (15.22)	607 (21.69)	1090 (38.68)	
Other Race	1102 (39.27)	929 (33.12)	756 (27.02)	585 (20.76)	
**Marital status, n (%)**					< 0.001
Married/ cohabiting	1560 (55.60)	1591 (56.72)	1543 (55.15)	1470 (52.16)	
Widowed/divorced/separated	412 (14.68)	322 (11.48)	319 (11.40)	327 (11.60)	
Never married	552 (19.67)	664 (23.67)	719 (25.70)	795 (28.21)	
Unclear	282 (10.05)	228 (8.13)	217 (7.76)	226 (8.02)	
**Education, n (%)**					< 0.001
Under high school	538 (19.17)	476 (16.97)	481 (17.19)	408 (14.48)	
High school or equivalent	545 (19.42)	526 (18.75)	582 (20.80)	585 (20.76)	
Above high school	1441 (51.35)	1574 (56.11)	1518 (54.25)	1599 (56.74)	
Unclear	282 (10.05)	229 (8.16)	217 (7.76)	226 (8.02)	
**PIR, n (%)**					0.042
≤ 1.26	813 (28.97)	730 (26.02)	748 (26.73)	704 (24.98)	
1.27–3.24	822 (29.29)	831 (29.63)	851 (30.41)	836 (29.67)	
≥ 3.25	936 (33.36)	1001 (35.69)	962 (34.38)	1052 (37.33)	
Unclear	235 (8.37)	243 (8.66)	237 (8.47)	226 (8.02)	
**BMI (mean ± SD, kg/m** ^**2**^ **)**	26.87 ± 6.40	28.18 ± 6.72	28.99 ± 6.84	29.79 ± 6.63	< 0.001
**Activity, n (%)**					< 0.001
Active	1716 (61.15)	1555 (55.44)	1466 (52.39)	1430 (50.75)	
Less active	171 (6.09)	197 (7.02)	228 (8.15)	204 (7.24)	
Inactive	913 (32.54)	1044 (37.22)	1097 (39.21)	1177 (41.77)	
Unclear	6 (0.21)	9 (0.32)	7 (0.25)	7 (0.25)	
**MET scores (median, IQR)**	120.00 (60.00-257.50)	130.00 (60.00-300.00)	150.00 (60.00-360.00)	180.00 (80.00-360.00)	< 0.001
**Smoking, n (%)**					< 0.001
Never	898 (32.00)	946 (33.73)	808 (28.88)	861 (30.55)	
Former	196 (6.99)	196 (6.99)	232 (8.29)	232 (8.23)	
Current	270 (9.62)	288 (10.27)	335 (11.97)	324 (11.50)	
Unclear	1442 (51.39)	1375 (49.02)	1423 (50.86)	1401 (49.72)	
**Alcohol, n (%)**					< 0.001
Yes	1675 (59.69)	1860 (66.31)	1975 (70.59)	2071 (73.49)	
No	37 (1.32)	42 (1.50)	64 (2.29)	44 (1.56)	
Unclear	1094 (38.99)	903 (32.19)	759 (27.13)	703 (24.95)	
**Hypertension, n (%)**					< 0.001
Yes	650 (23.16)	591 (21.07)	666 (23.80)	815 (28.92)	
No	2156 (76.84)	2214 (78.93)	2132 (76.20)	2003 (71.08)	
**Diabetes, n (%)**					0.045
Yes	270 (9.62)	278 (9.91)	265 (9.47)	324 (11.50)	
No	2536 (90.38)	2527 (90.09)	2533 (90.53)	2494 (88.50)	
**Thyroid disease, n (%)**					< 0.001
Yes	235 (8.37)	168 (5.99)	143 (5.11)	108 (3.83)	
No	2284 (81.40)	2401 (85.60)	2435 (87.03)	2481 (88.04)	
Unclear	287 (10.23)	236 (8.41)	220 (7.86)	229 (8.13)	
**Vitamin D intake (median (IQR), mcg)**	3.00 (1.45–5.45)	3.25 (1.55–5.80)	3.25 (1.55–5.80)	3.50 (1.65–6.30)	0.002
**Vitamin D supplements (median (IQR), (mcg)**	25.00 (10.00–35.00)	25.00 (10.00–35.00)	20.00 (10.05-30.00)	20.00 (10.00-26.48)	0.736
**Calcium intake (median (IQR), mg)**	792.50 (536.50-1087.25)	834.50 (570.50-1156.25)	876.50 (612.00-1226.75)	921.25 (636.50-1274.50)	< 0.001
**Calcium supplements (median (IQR), mg)**	333.00 (200.00-535.00)	220.00 (200.00-500.00)	220.00 (200.00-500.00)	210.00 (200.00-500.00)	< 0.001
**Vitamin D (mean ± SD, nmol/L)**	60.85 ± 26.00	59.97 ± 24.29	59.62 ± 24.26	58.70 ± 24.31	0.015
**Total calcium (mean ± SD, mg/dL)**	9.37 ± 0.37	9.37 ± 0.34	9.37 ± 0.34	9.40 ± 0.35	0.006
**ALT (median (IQR), U/L)**	20.00 (15.00–28.00)	20.00 (15.00–28.00)	21.00 (16.00–30.00)	21.00 (16.00–30.00)	0.026
**Total Cholesterol (mean ± SD, mg/dL)**	191.63 ± 39.68	188.38 ± 41.21	187.19 ± 39.61	185.22 ± 41.59	< 0.001
**Serum uric acid (mean ± SD, mg/dL)**	5.00 ± 1.30	5.21 ± 1.34	5.41 ± 1.39	5.67 ± 1.39	< 0.001
**Visceral fat mass (median (IQR),kg)**	0.44 (0.26–0.66)	0.42 (0.26–0.62)	0.43 (0.27–0.62)	0.40 (0.25–0.59)	< 0.001
**Lean body mass (mean ± SD,kg)**	44.10 ± 10.40	49.30 ± 11.35	53.22 ± 11.58	59.67 ± 12.16	< 0.001
**Log LM/VFM**	2.00 (1.85–2.19)	2.06 (1.91–2.25)	2.09 (1.94–2.28)	2.17 (2.02–2.35)	< 0.001
**Total BMD (mean ± SD,g/cm** ^**2**^ **)**	0.98 ± 0.05	1.08 ± 0.02	1.14 ± 0.02	1.26 ± 0.06	< 0.001

Abbreviations:

*BMD* bone mineral density, *SD* standard deviation, *IQR* interquartile range, *PIR* ratio of family income to poverty, *BMI* body mass index, *ALT* alanine aminotransferase, *LM/VFM* lean body mass/ visceral fat mass, *MET* metabolic equivalent

### Association of lean body mass and visceral fat mass with BMD

The findings from the regression analysis are presented in Table 2. The results indicate that after adjusting for covariates, lean body mass exhibited a positive association with BMD, both in its continuous form (β = 0.078; 95% CI: 0.074, 0.082) and when converted to a categorical variable. Specifically, the highest quartile of total lean mass was related to higher BMD values (β = 0.138; 95% CI: 0.129, 0.148). In contrast, visceral fat mass demonstrated a negative association with BMD when analyzed as a continuous variable (β = -0.027; 95% CI: -0.030, -0.024) and when considered as the highest quartile of a categorical variable (β = -0.065; 95% CI: -0.072, -0.057).

**Table 2:** Multiple linear regression analysis for the relationship between lean body mass, visceral fat mass and lean body mass to visceral fat ratio with bone mineral density in the National Health and Nutrition Examination Survey 2011–2018

Independent variables	Crude Model		R^2^	Model I		R^2^	Model II		R^2^
	β (95%CI)	P-value		β (95%CI)	P-value		β (95%CI)	P-value	
**Total lean mass**									
Per-SD increase	0.050 (0.048, 0.052)	< 0.001	0.218	0.047 (0.045, 0.049)	< 0.001	0.252	0.078 (0.074, 0.082)	< 0.001	0.302
Q1	Reference			Reference			Reference		
Q2	0.048 (0.043, 0.053)	< 0.001	0.200	0.043 (0.037, 0.048)	< 0.001	0.235	0.050 (0.044, 0.056)	< 0.001	0.269
Q3	0.081 (0.076, 0.086)	< 0.001		0.071 (0.065, 0.077)	< 0.001		0.086 (0.079, 0.094)	< 0.001	
Q4	0.129 (0.124, 0.134)	< 0.001		0.117 (0.111, 0.124)	< 0.001		0.138 (0.129, 0.148)	< 0.001	
*P* for trend	< 0.001			< 0.001			< 0.001		
**Visceral fat mass**									
Per-SD increase	-0.001 (-0.003, 0.000)	0.128	0.001	-0.001 (-0.003, 0.001)	0.377	0.149	-0.027 (-0.030, -0.024)	< 0.001	0.237
Q1	Reference			Reference			Reference		
Q2	0.005 (-0.001, 0.010)	0.099	0.002	0.002 (-0.004, 0.007)	0.552	0.150	-0.021 (-0.026, -0.015)	< 0.001	0.234
Q3	0.007 (0.002, 0.013)	0.011		0.007 (0.002, 0.013)	0.013		-0.032 (-0.038, -0.025)	< 0.001	
Q4	-0.005 (-0.011, 0.000)	0.062		-0.004 (-0.010, 0.002)	0.203		-0.065 (-0.072, -0.057)	< 0.001	
*P* for trend	< 0.001			0.151			< 0.001		
**LogLM/VFM**									
Per-SD increase	0.022 (0.020, 0.024)	< 0.001	0.041	0.016 (0.014, 0.018)	< 0.001	0.164	0.034 (0.032, 0.037)	< 0.001	0.260
Q1	Reference			Reference			Reference		
Q2	0.038 (0.032, 0.043)	< 0.001	0.046	0.025 (0.020, 0.030)	< 0.001	0.163	0.036 (0.032, 0.042)	< 0.001	0.253
Q3	0.049 (0.044, 0.054)	< 0.001		0.032 (0.027, 0.038)	< 0.001		0.056 (0.050, 0.062)	< 0.001	
Q4	0.060 (0.054, 0.065)	< 0.001		0.041 (0.035, 0.047)	< 0.001		0.084 (0.076, 0.091)	< 0.001	
*P* for trend	< 0.001			< 0.001			< 0.001		

*Abbreviations*: *BMD* bone mineral density, *β* partial regression coefficient, *CI* confidence interval, *LM/VFM* Lean body mass/Visceral fat mass

Crude Model: no covariates were adjusted. Model 1: gender, age and race were adjusted. Model 2: gender, age, marital status, race, education, PIR, BMI, activity, MET scores, smoking, alcohol, hypertension, diabetes, thyroid disease, serum vitamin D, total cholesterol, alanine aminotransferase, total calcium, serum uric acid, vitamin D intake, vitamin D supplements, calcium intake, and calcium supplements

The analysis of LogLM/VFM as the sole exposure variable revealed a significant positive association with BMD, both in terms of a continuous variable (β = 0.034; 95% CI: 0.032, 0.037) and a categorical variable. A favorable relationship was noted for the highest quartile (β = 0.084; 95% CI: 0.076, 0.091). The P for trend values for all variables of model 2 showed statistical differences.

### Subgroup analysis

The results of the subgroup analyses based on gender, age, BMI, hypertension, and diabetes are displayed in Table 3. The analyses demonstrated a consistent positive correlation between total lean mass and BMD in all subgroups. In contrast, a negative association between visceral fat mass and BMD was observed except in diabetic patients. Furthermore, Log LM/VFA showed a clear and significant correlation with BMD when investigated as an independent exposure variable.

**Table 3:** The subgroup analysis of the associations between lean body mass, visceral fat mass and lean body mass to visceral fat ratio with bone mineral density in the National Health and Nutrition Examination Survey 2011–2018

Independent variables	Per-SD increase β (95% CI)	Quartile β (95% CI)			
		Q1	Q2	Q3	Q4
**Total lean mass**					
Stratified by gender					
Male	0.086 (0.081, 0.092) ^a^	Reference	0.057 (0.040, 0.075) ^a^	0.097 (0.080, 0.115) ^a^	0.153 (0.134, 0.171) ^a^
Female	0.082 (0.076, 0.089) ^a^	Reference	0.054 (0.048, 0.061) ^a^	0.091 (0.080, 0.101)^a^	0.108 (0.092, 0.124) ^a^
Stratified by age					
<50	0.079 (0.075, 0.084) ^a^	Reference	0.050 (0.044, 0.056) ^a^	0.087 (0.079, 0.095) ^a^	0.139 (0.129, 0.150) ^a^
≥50	0.074 (0.065, 0.084) ^a^	Reference	0.052 (0.039, 0.066) ^a^	0.080 (0.063, 0.098) ^a^	0.133 (0.111, 0.156) ^a^
BMI					
<25	0.091 (0.083, 0.098) ^a^	Reference	0.056 (0.046, 0.066) ^a^	0.099 (0.087, 0.112) ^a^	0.153 (0.133, 0.173) ^a^
25–30	0.082 (0.074, 0.089) ^a^	Reference	0.052 (0.042, 0.062) ^a^	0.094 (0.079, 0.109) ^a^	0.145 (0.129, 0.162) ^a^
≥30	0.044 (0.039, 0.049) ^a^	Reference	0.040 (0.026, 0.055) ^a^	0.064 (0.048, 0.079) ^a^	0.107 (0.090, 0.124) ^a^
Stratified by hypertension					
Yes	0.077 (0.068, 0.085) ^a^	Reference	0.053 (0.038, 0.067) ^a^	0.083 (0.067, 0.100) ^a^	0.131 (0.110, 0.152) ^a^
No	0.082 (0.077, 0.086) ^a^	Reference	0.053 (0.047, 0.060) ^a^	0.092 (0.084, 0.101) ^a^	0.146 (0.135, 0.156) ^a^
Stratified by diabetes					
Yes	0.068 (0.056, 0.081) ^a^	Reference	0.065 (0.043, 0.087) ^a^	0.095 (0.070, 0.120) ^a^	0.135 (0.104, 0.167) ^a^
No	0.083 (0.078, 0.087) ^a^	Reference	0.051 (0.045, 0.058) ^a^	0.089 (0.081, 0.097) ^a^	0.143 (0.133, 0.154) ^a^
**Visceral fat mass**					
Stratified by gender					
Male	-0.036 (-0.040, -0.031) ^a^	Reference	-0.025 (-0.033, -0.017) ^a^	-0.042 (-0.052, -0.032) ^a^	-0.082 (-0.094, -0.070) ^a^
Female	-0.022 (-0.026, -0.017) ^a^	Reference	-0.014 (-0.022, -0.007) ^a^	-0.025 (-0.033, -0.016) ^a^	-0.048 (-0.059, -0.037) ^a^
Stratified by age					
<50	-0.029 (-0.032, -0.025) ^a^	Reference	-0.019 (-0.025, -0.013) ^a^	-0.030 (-0.037, -0.023) ^a^	-0.064 (-0.072, -0.055) ^c^
≥50	-0.022 (-0.028, -0.016) ^a^	Reference	-0.021 (-0.039, -0.003) ^a^	-0.031 (-0.049, -0.013) ^a^	-0.054 (-0.073, -0.035) ^a^
BMI					
<25	-0.030 (-0.038, -0.022) ^a^	Reference	-0.015 (-0.022, -0.007) ^a^	-0.034 (-0.046, -0.023) ^a^	-0.075 (-0.098, -0.052) ^a^
25–30	-0.036 (-0.043, -0.030) ^a^	Reference	-0.031 (-0.042, -0.019) ^a^	-0.050 (-0.062, -0.038) ^a^	-0.082 (-0.097, -0.068) ^a^
≥30	-0.017 (-0.020, -0.013) ^a^	Reference	-0.030 (-0.048, -0.012) ^c^	-0.035 (-0.052, -0.017) ^a^	-0.059 (-0.077, -0.041) ^a^
Stratified by hypertension					
Yes	-0.019 (-0.024, -0.014) ^a^	Reference	-0.014 (-0.031, 0.003)	-0.022 (-0.039, -0.005) ^c^	-0.045 (-0.064, -0.026) ^a^
No	-0.034 (-0.037, -0.030) ^a^	Reference	-0.022 (-0.028, -0.016) ^a^	-0.037 (-0.044, -0.030) ^a^	-0.072 (-0.081, -0.063) ^a^
Stratified by diabetes					
Yes	-0.005 (-0.012, 0.002)	Reference	0.021 (-0.017, 0.058)	0.014 (-0.022, 0.049)	0.011 (-0.026, 0.047)
No	-0.032 (-0.035, -0.028) ^a^	Reference	-0.023 (-0.029, -0.018) ^a^	-0.036 (-0.043, -0.029) ^a^	-0.072 (-0.081, -0.064) ^a^
**LogLM/VFM**					
Stratified by gender					
Male	0.053 (0.049, 0.058) ^a^	Reference	0.046 (0.038, 0.054) ^a^	0.077 (0.068, 0.085) ^a^	0.112 (0.102, 0.123) ^a^
Female	0.024 (0.021, 0.027) ^a^	Reference	0.030 (0.023, 0.037) ^a^	0.039 (0.032, 0.047) ^a^	0.062 (0.053, 0.071) ^a^
Stratified by age					
<50	0.030 (0.028, 0.033) ^a^	Reference	0.029 (0.024, 0.035) ^a^	0.050 (0.044, 0.056) ^a^	0.077 (0.069, 0.084) ^a^
≥50	0.035 (0.029, 0.041) ^a^	Reference	0.042 (0.031, 0.052)	0.051 (0.038, 0.064) ^a^	0.073 (0.055, 0.092) ^a^
BMI					
<25	0.026 (0.022, 0.030) ^a^	Reference	0.029 (0.014, 0.044) ^a^	0.048 (0.033, 0.062) ^a^	0.076 (0.060, 0.091) ^a^
25–30	0.045 (0.040, 0.050) ^a^	Reference	0.047 (0.038, 0.056) ^a^	0.067 (0.058, 0.077) ^a^	0.101 (0.088, 0.114) ^a^
≥30	0.041 (0.036, 0.046) ^a^	Reference	0.032 (0.024, 0.040) ^a^	0.055 (0.046, 0.065) ^a^	0.089 (0.074, 0.103) ^a^
Stratified by hypertension					
Yes	0.036 (0.030, 0.042) ^a^	Reference	0.045 (0.035, 0.055) ^a^	0.058 (0.046, 0.071) ^a^	0.077 (0.060, 0.094) ^a^
No	0.034 (0.031, 0.037) ^a^	Reference	0.032 (0.026, 0.038) ^a^	0.055 (0.048, 0.061) ^a^	0.084 (0.077, 0.092) ^a^
Stratified by diabetes					
Yes	0.022 (0.012, 0.032) ^a^	Reference	0.018 (0.003, 0.034) ^c^	0.030 (0.008, 0.051) ^b^	0.019 (-0.014, 0.053)
No	0.036 (0.033, 0.039) ^a^	Reference	0.040 (0.034, 0.045) ^a^	0.060 (0.054, 0.066) ^a^	0.090 (0.083, 0.098) ^a^

*Abbreviations*: *β* partial regression coefficient, *CI* confidence interval, *BMD* bone mineral density, *LM/VFM* lean body mass/visceral fat mass

All variables including gender, age and race were adjusted. Model 2: gender, age, marital status, race, education, PIR, BMI, activity, MET scores, smoking, alcohol, hypertension, diabetes, thyroid disease, serum vitamin D, total cholesterol, alanine aminotransferase, total calcium, serum uric acid, vitamin D intake, vitamin D supplements, calcium intake, and calcium supplements were adjusted for, except for the stratification variable which was not adjusted for

^a^ P < 0.001, ^b^ P < 0.01, ^c^ P < 0.05

### Smoothing curve fitting and analysis of threshold and saturation effects

**Fig. 2:**
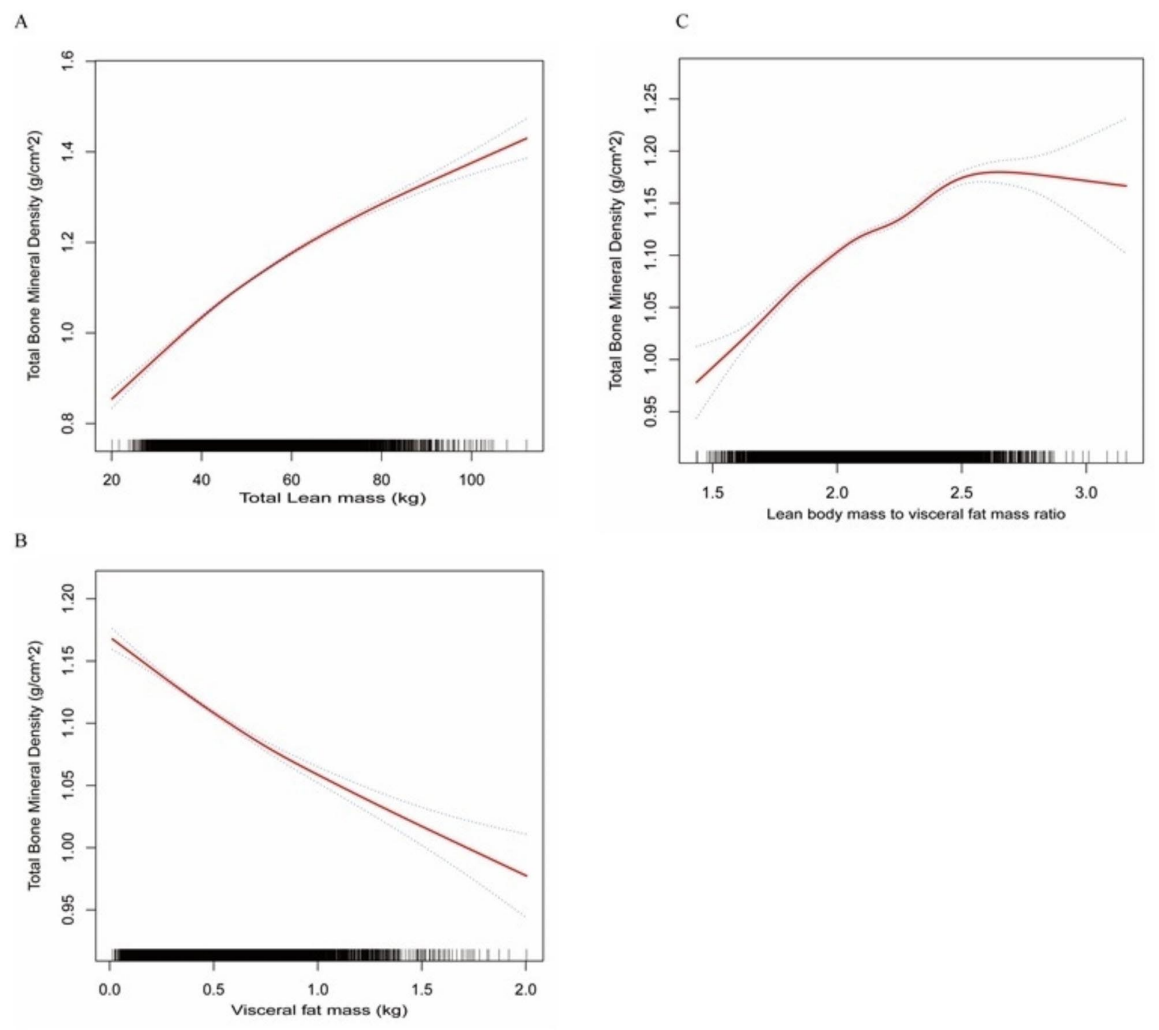
Adjusted dose-response relationships between lean body mass (**A**), visceral fat mass (**B**), and lean body mass to visceral fat ratio (**C**) with bone mineral density in the National Health and Nutrition Examination Survey 2011–2018. Adjusted for gender, age, marital status, race, education, PIR, BMI, activity, MET scores, smoking, alcohol, hypertension, diabetes, thyroid disease, serum vitamin D, total cholesterol, alanine aminotransferase, total calcium, serum uric acid, vitamin D intake, vitamin D supplements, calcium intake, and calcium supplements

The smoothing curve fitting analysis showed a weak positive non-linear trend between total lean mass and BMD (Fig. 2a). In contrast, visceral fat mass displayed a weaker non-linear negative trend with BMD (Fig. 2b). Remarkably, the independent exposure variable analysis of Log LM/VFM demonstrated a significant positive non-linear trend with BMD (Fig. 2c), which reached an inflection point of 2.60 (Table 4). When the Log LM/VFM was < 2.60, the BMD increased with an adjusted β of 0.14(95% CI: 0.13 0.16 P < 0.001) for the per-unit increase in the Log LM/VFM. When the value is beyond 2.60, the association did not show a statistical difference of (β = -0.08; 95% CI: -0.17, 0.01).

**Table 4:** Threshold effect analysis of lean body mass to visceral fat mass ratio with bone mineral density using piece-wise linear regression in the National Health and Nutrition Examination Survey 2011–2018

Inflection point	Effect size (β)	95%CI	P-value
Model I			
One line effect	0.14	(0.13, 0.16)	< 0.001
Model II			
Log LM/VFM < 2.6	0.15	(0.14, 0.17)	< 0.001
Log LM/VFM ≥ 2.6	-0.08	(-0.17, 0.01)	0.095
Log-likelihood ratio test			< 0.001

*Abbreviations*: *β* partial regression coefficient, *CI* confidence interval, *LM/VFM* lean body mass/visceral fat mass

Adjusted for gender, age, marital status, race, education, PIR, BMI, activity, MET scores, smoking, alcohol, hypertension, diabetes, thyroid disease, serum vitamin D, total cholesterol, alanine aminotransferase, total calcium, serum uric acid, vitamin D intake, vitamin D supplements, calcium intake, and calcium supplements

## Discussion

In a large-scale population study based on the NHANES database, we found a positive correlation between lean body mass and BMD, while visceral fat mass was negatively correlated. We used Log LM/VFM as an alternative estimate to understand the combined effect on BMD. The results showed a positive correlation between Log LM/VFM and BMD, with an increase in BMD as LogLM/VFM increased. In regression analysis, to minimize potential bias, we extensively considered possible confounding factors to ensure the reliability of the results. At the same time, we further conducted a stratified analysis based on gender, age, BMI, hypertension, and diabetes. Except for the diabetic population, the subgroup analysis of all populations showed statistical significance, and it should be noted that this may be related to the small sample size of the diabetic population. These results all indicate that the conclusion has a considerable degree of robustness. In further analysis of smooth curve fitting and threshold effects, we found a nonlinear trend and threshold effect between LogLM/VFM and BMD.

Lean body mass refers to the body’s total weight, excluding adipose tissue or fat. One of the critical components of lean mass is skeletal muscle, which is responsible for movement and stability. Despite being a passive structural element, skeletal muscles function as an endocrine organ that releases an extensive range of muscle factors known as myokines, such as insulin-like growth factor-1, fibroblast growth factor-2, brain-derived neurotrophic factor et al., which play a role in regulating other cells in the body [27]. These muscle factors actively participate in bone metabolism by interacting with the osteoblasts or osteoclasts. Previous research has established a positive correlation between lean mass and BMD. Ilesanmi-Oyelere et al. demonstrated that BMD was more strongly associated with lean body mass than fat mass [28]. Likewise, Xiao’s study revealed that appendicular lean mass was a robust predictor of BMD in both genders [29]. Our study excluded bone mineral content from assessing lean body mass to avoid potential interference. The results showed a positive association between lean body mass and BMD, and this relationship did not vary according to the various characteristics of the study participants.

Researchers have investigated the relationship between adipose tissue and BMD with inconsistent results. While some studies suggest that obesity may have a protective effect on bone metabolism, as individuals with obesity generally exhibit higher BMD [30], others present a contrasting view. A positive correlation between abdominal fat and peripheral BMD and microstructure was established in Liu et al.‘s study. However, this correlation becomes insignificant once the researchers adjust BMI or weight [31]. Moreover, Freitas et al.‘s study posit that overall fat mass can be a protective factor against osteoporosis [32]. However, other studies have found the opposite. Zhang et al.‘s study found that the larger the visceral fat area, the lower the BMD, and the higher the risk of vertebral compression fractures [33]. This study’s findings concur with Zhu et al.‘s research, which establishes a negative correlation between visceral fat area and BMD regardless of subjects with average BMI or obesity [13].

These inconsistencies may stem from variations in the influence of fat tissue in different body parts on BMD. Whereas subcutaneous or total fat [34] may have a protective effect on BMD, central or visceral fat accumulation may have a detrimental effect [29, 35]. Excessive deposition of visceral fat tissue could lead to the release of inflammatory factors such as resistin, tumor necrosis factor-α, and interleukin-6 and a decrease in anti-inflammatory factors like adiponectin [36], which could eventually enhance osteoclast activity and suppress bone formation [37, 38]. Additionally, excessive visceral fat results in lipid metabolism disturbance leading to an increase in free fatty acids [39], which are lipotoxic to osteoblasts and osteoclasts, resulting in a higher risk of low bone mass [40].

The complexity of the association between muscle and fat tissues in the human body makes it challenging to comprehend. Lee et al. [41] analyzed 2507 individuals based on Mendelian randomization in the Korean Genome Epidemiology Study showed that BMD increased with lean body mass in both men and women. In contrast, a positive association of fat mass with BMD was observed only in men and premenopausal women. Unlike the former study, Jain et al. [42] studied the American population using NHANES data, and they found a negative correlation between fat mass and BMD. In contrast, lean body mass was positively correlated with BMD. In general, existing research lacks a holistic perspective on the joint effects of lean body mass and adipose tissue. In addition, previous studies have used those metrics without considering the role of different adipose tissues in the body, particularly visceral adipose tissue. Therefore, we used the new metric of lean body mass to visceral fat mass ratio to more accurately correlate the ratio of the two compositions with BMD.

Independently, lean mass positively correlated with BMD, whereas visceral fat mass was inversely related. However, a non-linear pattern with a threshold was recognized when LogLM/VFM was used. Log LM/VFM showed a significant positive association with BMD when it had values of less than 2.60. This association implies that maintaining optimal BMD values necessitates elevated lean mass or lower amounts of visceral fat mass. However, maintaining a balanced ratio of lean to visceral fat mass is critical, and exceeding the threshold of 2.60 will not improve BMD levels further. Overall, this research stresses the intricacy of the relationships between muscle, fat deposits, and BMD, emphasizing the need to maintain a balanced and healthy lifestyle to maintain optimal bone health.

The study has a few limitations that must be acknowledged. Firstly, it is a cross-sectional study, which restricts the ability to make conclusive causal inferences. Thus, further population cohort studies are needed to validate the findings of this study. Secondly, cross-sectional studies are susceptible to confounding variables that may influence the results. Despite our efforts to consider potential factors, there may be unknown confounding factors, such as the subject’s parathyroid hormone (PTH) level that could impact the results. Unfortunately, due to data limitations, we could not obtain the PTH levels of the subjects in this study. Although we included a surrogate indicator of thyroid disease as a confounding variable in the multivariate analysis, it cannot fully substitute the effect of hormone levels on BMD, which may introduce bias to the analysis results. Additionally, the level of outdoor activity can also affect BMD. While we accounted for the intensity of daily activities of the study subjects, these indicators cannot wholly replace the impact of outdoor activities on BMD. Accordingly, the results of this study need to be treated with caution, and future research needs to consider the possible impact of these factors further. In addition, BMD based on dual-energy X-ray measurement is two-dimensional and cannot evaluate changes in the microstructure of bones. Therefore, we cannot make further correlation analysis.

In conclusion, the present study showed a positive association between lean body mass and BMD, while visceral fat mass and BMD were found to be negatively associated. In a thorough analysis of the relationship between the two and BMD using LogLM/VFM as a proxy variable, we found a nonlinear positive relationship with a threshold effect. Log LM/VFM has value beyond evaluating lean body mass or visceral fat mass alone.

## Data Availability

All data for this study are publicly available from the following websites: https://www.cdc.gov/nchs/nhanes/about_nhanes.htm.
